# Tropisms of AAV for Subretinal Delivery to the Neonatal Mouse Retina and Its Application for *In Vivo* Rescue of Developmental Photoreceptor Disorders

**DOI:** 10.1371/journal.pone.0054146

**Published:** 2013-01-15

**Authors:** Satoshi Watanabe, Rikako Sanuki, Shinji Ueno, Toshiyuki Koyasu, Toshiaki Hasegawa, Takahisa Furukawa

**Affiliations:** 1 Laboratory for Molecular and Developmental Biology, Institute for Protein Research, Osaka University, Suita, Osaka, Japan; 2 JST, CREST, Suita, Osaka, Japan; 3 Department of Developmental Biology, Osaka Bioscience Institute, Suita, Osaka, Japan; 4 Kyoto University Graduate School of Medicine, Sakyo-ku, Kyoto, Kyoto, Japan; 5 Department of Ophthalmology, Nagoya University Graduate School of Medicine, Showa-ku, Nagoya, Aichi, Japan; 6 Research Center for Ultra-high Voltage Electron Microscopy, Osaka University, Ibaraki, Osaka, Japan; University of Florida, United States of America

## Abstract

**Background:**

Adeno-associated virus (AAV) is well established as a vehicle for *in vivo* gene transfer into the mammalian retina. This virus is promising not only for gene therapy of retinal diseases, but also for *in vivo* functional analysis of retinal genes. Previous reports have shown that AAV can infect various cell types in the developing mouse retina. However, AAV tropism in the developing retina has not yet been examined in detail.

**Methodology/Principal Findings:**

We subretinally delivered seven AAV serotypes (AAV2/1, 2/2, 2/5, 2/8, 2/9, 2/10, and 2/11) of AAV-CAG-mCherry into P0 mouse retinas, and quantitatively evaluated the tropisms of each serotype by its infecting degree in retinal cells. After subretinal injection of AAV into postnatal day 0 (P0) mouse retinas, various retinal cell types were efficiently transduced with different AAVs. Photoreceptor cells were efficiently transduced with AAV2/5. Retinal cells, except for bipolar and Müller glial cells, were efficiently transduced with AAV2/9. Horizontal and/or ganglion cells were efficiently transduced with AAV2/1, AAV2/2, AAV2/8, AAV2/9 and AAV2/10. To confirm the usefulness of AAV-mediated gene transfer into the P0 mouse retina, we performed AAV-mediated rescue of the *Cone-rod homeobox* gene knockout (*Crx* KO) mouse, which exhibits an outer segment formation defect, flat electroretinogram (ERG) responses, and photoreceptor degeneration. We injected an AAV expressing *Crx* under the control of the *Crx 2kb* promoter into the neonatal *Crx* KO retina. We showed that AAV mediated-Crx expression significantly decreased the abnormalities of the *Crx* KO retina.

**Conclusion/Significance:**

In the current study, we report suitable AAV tropisms for delivery into the developing mouse retina. Using AAV2/5 in photoreceptor cells, we demonstrated the possibility of gene replacement for the developmental disorder and subsequent degeneration of retinal photoreceptors caused by the absence of *Crx*.

## Introduction

Functional analysis of the genes expressed in the mammalian retina is essential for understanding the molecular basis for human retinal development and disease. Recent progress in techniques of comprehensive analysis of gene expression using microarray and next generation sequencing make it possible to obtain many candidate genes that are possibly associated with retinal development and disease [1], [2]. Although *in vivo* analysis of candidate genes using transgenic and/or knockout mice is beneficial in revealing the *in vivo* functions of the genes, it is still expensive, time-consuming, and requires a great deal of skill. Therefore, a rapid and convenient method of *in vivo* gene transfer would be beneficial to the field. To transduce a gene into the mouse retina, *in vivo* electroporation and virus-mediated gene transfer are currently the most available methods. *In vivo* electroporation is a method in which plasmid DNA is incorporated into retinal tissue by high-voltage pulses. This method efficiently transduces DNA into rod photoreceptor cells, but much less efficiently into bipolar, amacrine, and Müller glial cells. Moreover, cone photoreceptor, horizontal, and ganglion cells are barely transduced by *in vivo* electroporation [3]. For virus-mediated transduction, retrovirus, lentivirus, adenovirus, and adeno-associated virus (AAV) have been developed as vehicles for retinal gene transfer. In particular, AAV has many advantages for retinal gene transfer, including high transduction efficiency in non-dividing cells, long-term transgene expression, and low-toxicity. AAV is a non-pathogenic parvovirus, which consists of single-stranded DNA covered with capsid proteins. Each AAV serotype is different in the capsid structure, which leads to different tropisms and transduction efficiencies. Twelve serotypes have currently been used as a vehicle for *in vivo* gene transfer (AAV2/1-AAV2/12). AAV tropisms for gene transduction into several murine organs and tissues, including the retina, are different according to developmental stage (neonatal or adult) [4], [5]. The previous studies on AAV serotype tropism in subretinal injections into the adult mouse retina revealed that retinal pigment epithelium (RPE) cells are efficiently transduced with AAV2/1, and RPE and photoreceptor cells are efficiently transduced with AAV2/2, AAV2/5 [6], [7], and AAV2/8 [7]. However, detailed AAV tropisms for transduction into the developing mouse retina have not been reported.

In the current study, we examined the tropism of seven AAV serotypes (AAV2/1, AAV2/2, AAV2/5, AAV2/8, AAV2/9, AAV2/10, and AAV2/11) by subretinal injection into the P0 mouse retina. We revealed that AAV can transduce encoded genes into various retinal cell types in the developing mouse retina. In addition, to validate the usefulness of AAV-mediated gene transfer into the developing mouse retina, we performed AAV-mediated rescue of *Crx* KO mice. CRX is a transcription factor that is predominantly expressed in photoreceptor cells and is essential for photoreceptor maturation [8], [9], [10]. We previously reported that *Crx* KO mice exhibit a total lack of outer segment formation, an absence of both scotopic and photopic electroretinograms (ERG), and progressive photoreceptor degeneration [10]. Our AAV-mediated rescue experiment led to a partial restoration of morphological and functional characteristics in the *Crx* KO retina. In humans, the mutations of *Crx* are associated with three forms of retinal degeneration, including cone and rod dystrophy (CORD) [11], [12], [13], retinitis pigmentosa (RP) [13], and Leber congenital amaurosis (LCA) [13], [14], all of which can lead to vision loss. Thus, our results also provide a clue to the suitability of gene therapy for development disorders and degeneration of the retina in humans.

## Results

### Tropisms of Seven AAV Serotypes to the Neonatal Mouse Retina

**Figure 1:**
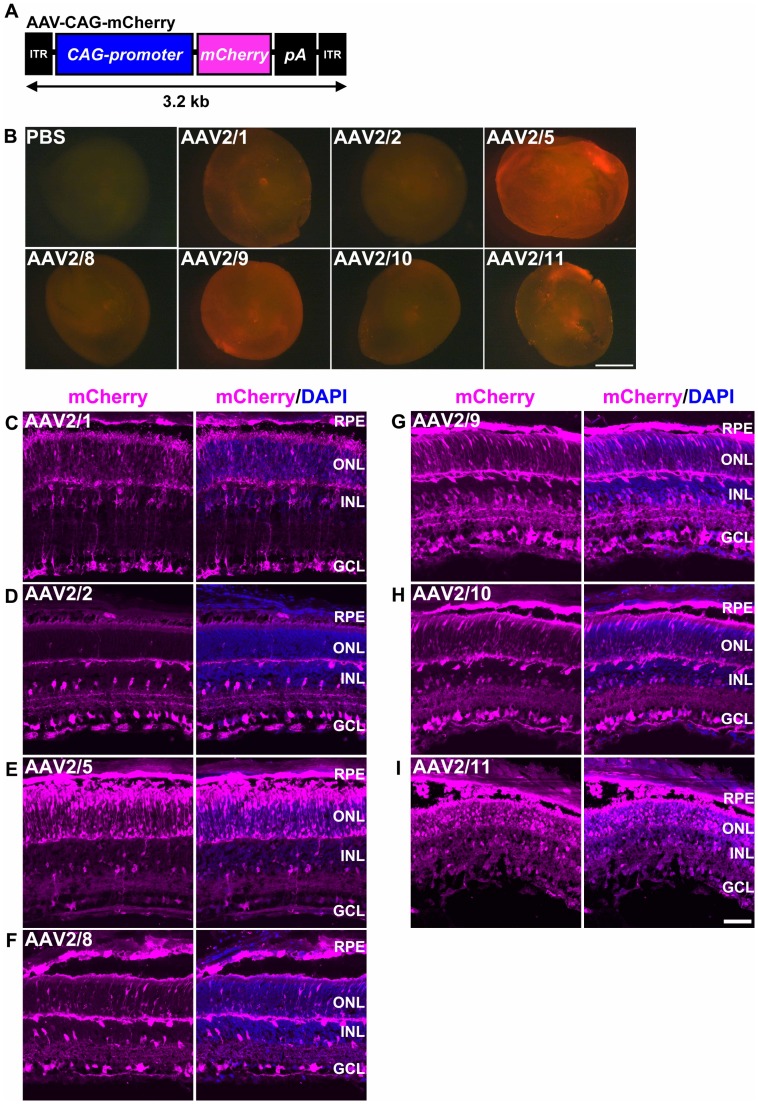
Tropisms of seven AAV serotypes in the P0 mouse retina. (A) Schematic diagram of the AAV-CAG-mCherry construct. This AAV drives ubiquitous expression of mCherry under the control of the *CAG* promoter. (B) Fluorescence images of the distribution of mCherry expression in whole retinas (photoreceptor-side-up) two weeks after subretinal injection with PBS or one of each of the seven serotypes of AAV-CAG-mCherry. (C–I) Fluorescence images of mCherry expression two weeks after subretinal injection into the P0 mouse retina with AAV2/1- (C), AAV2/2- (D), AAV2/5- (E), AAV2/8- (F), AAV2/9 (G), AAV2/10- (H), and AAV2/11- (I), CAG-mCherry. Scale bar represents 1 mm (B) and 50 µm (C–I). ITR: inverted terminal repeat, RPE: retinal pigment epithelium, ONL: outer nuclear layer, INL: inner nuclear layer, GCL: ganglion cell layer.

In order to examine AAV tropisms for subretinal delivery into the P0 mouse retina, we generated AAV2/1-, AAV2/2-, AAV2/5-, AAV2/8-, AAV2/9-, AAV2/10-, and AAV2/11-vectors expressing *mCherry* driven by the ubiquitous promoter, *CAG* promoter (AAV-CAG-mCherry) (Fig. 1A). We selected six serotypes (AAV2/1, 2/2, 2/5, 2/8, 2/9, 2/10), because they are known to be infectious to the mammalian central nervous system (Gene therapy program at university of Pennsylvania (http://www.med.upenn.edu/gtp/)), and have previously been examined for tropisms for subretinal or intravitreal transduction into the adult mouse retina [6], [7], [15]. Since the AAV2/11 serotype was recently discovered [16], we also tested this serotype in addition to the other six. Each of the seven tested serotypes of AAV was subretinally injected into the P0 mouse retina. We harvested the injected retinas at 14 days after injection, P14, when all retinal cells had finished generating (Fig. 1B–I). We observed that mCherry expression was evenly distributed in the retinas injected with each of the seven serotypes of AAV-CAG-mCherry (Fig. 1B). AAV2/5- and AAV2/9-injected retinas showed intense mCherry signals throughout, and AAV2/2-, AAV2/8-, and AAV2/10-injected retinas showed substantial mCherry signals (Fig. 1B). Photoreceptor cells were efficiently transduced with AAV2/1, AAV2/5, AAV2/9, and AAV2/11 (Fig. 1C, E, G, I). In particular, the AAV2/5-injected retina showed mCherry expression predominantly in photoreceptor cells (Fig. 1E). The AAV2/9-injected retina showed mCherry expression throughout the retina (Fig. 1G). Horizontal cells were efficiently transduced with AAV2/1, AAV2/2, AAV2/8, AAV2/9, and AAV2/10 (Fig. 1C, D, F–H). Müller glial cells were efficiently transduced with AAV2/1 (Fig. 1C). In addition, the RPE was well transduced with all serotypes (Fig. 1C–I).

### Infection Efficiencies of Seven AAV Serotypes

**Figure 2:**
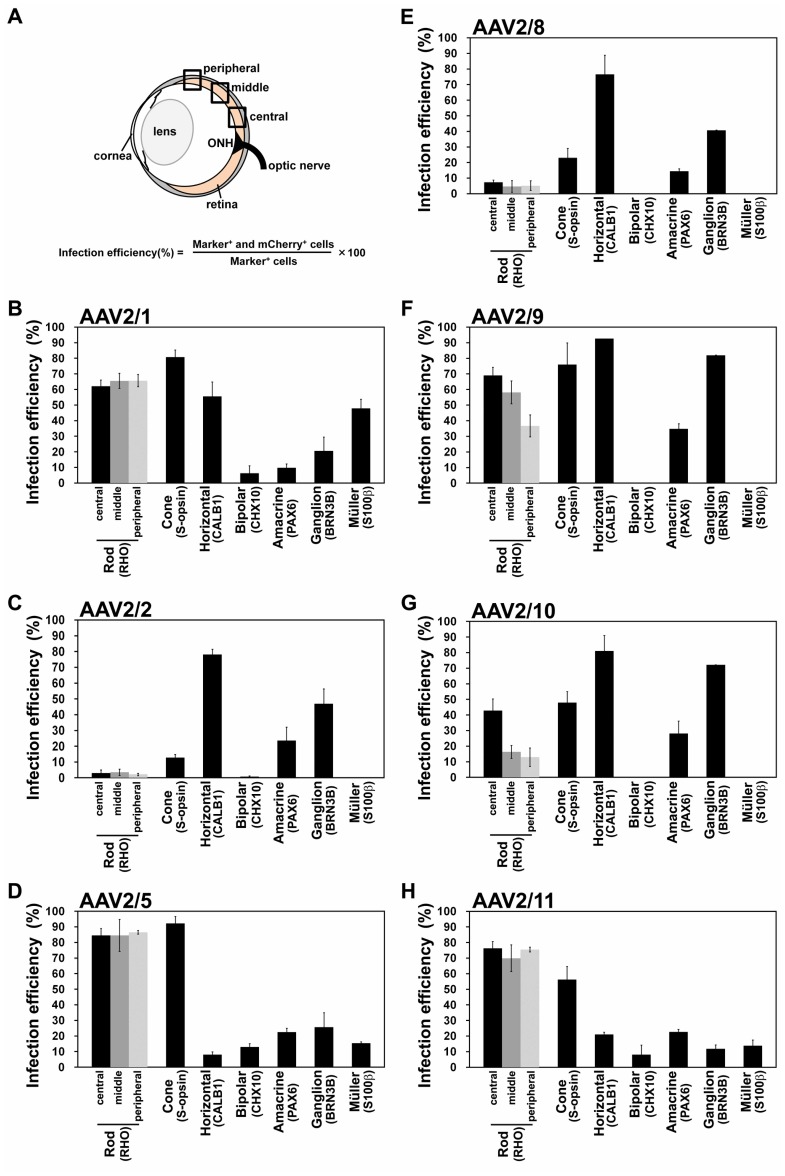
Infection efficiencies of seven AAV serotypes in each retinal cell type. (A) Schematic diagram of quantification method of infection efficiency. Two weeks after subretinal injection, the retinas were immunostained with antibodies of retinal cell type-specific makers (Rod: RHODOPSIN (RHO), Cone: S-OPSIN, Horizontal: CALB1, Bipolar: CHX10, Amacrine: PAX6, Ganglion: BRN3B, Müller: S100β). According to retinal cell types, the numbers of marker-positive and marker/mCherry double-positive cells were counted for calculation of infection efficiency (n = 3 from three different mice). Cells were counted in the central area of the retina for calculating the infection efficiencies of cone photoreceptor, bipolar, and Müller glial cells, and in the central, middle and peripheral areas of the retina for calculating the infection efficiencies of rod photoreceptor and amacrine cells. Infection efficiency was calculated using the formula indicated in Figure 2A. (B–H) Infection efficiencies of AAV2/1- (B), AAV2/2- (C), AAV2/5- (D), AAV2/8- (E), AAV2/9- (F), AAV2/10- (G), and AAV2/11- (H), CAG-mCherry. Error bar represents the SD from the means of three retinas. ONH: optic nerve head.

To quantitatively assess the tropism of seven AAV serotypes in the mouse retina, we measured the infection efficiencies for each retinal cell type. Infection efficiency is calculated by the percentage of cells expressing mCherry out of retinal cell-specific marker-positive cells (Fig. 2A). Photoreceptor and horizontal cells were efficiently transduced with AAV2/1 (Fig. 2B, Rod: 65.5±3.9% in the middle area, Cone: 80.8±4.5% and horizontal: 55.6±9.2%). The AAV2/1-injected retinas also exhibited the highest efficiency for Müller glial cells among seven serotypes (Fig. 2B, 47.9±5.8%). AAV2/2 and AAV2/8 showed similar transduction patterns with each other, and horizontal and ganglion cells were transduced mainly with these serotypes (Fig. 2C, E, horizontal cells: 78.2±3.2% and 76.6±12.2% respectively; ganglion cells: 46.9±9.4% and 40.7±0.1% respectively). AAV2/5 displayed the highest infection efficiency for rod and cone photoreceptor cells (Fig. 2D, rod; 84.5±10.3% in middle area and cone; 92.2±4.4%). Retinal cells except for bipolar and Müller glial cells were efficiently infected with AAV2/9, and infection efficiencies of AAV2/9 for horizontal and ganglion cells were the highest among seven serotypes (Fig. 2F, horizontal; 92.7±0.6% and ganglion; 82.0±0.1%). AAV2/9 showed the highest efficiency in amacrine cells (Fig. 2F, 34.8±3.2%). AAV 2/10 efficiently targeted horizontal and ganglion cells (Fig. 2G, horizontal cells: 81.0±9.9% and ganglion cells: 72.1±0.1%). AAV2/11 showed efficient infection in photoreceptor cells (Fig. 2H, rod: 70.0±8.6% in middle area and cone: 56.3±8.2%). Bipolar cells exhibited very low efficiency or no infection detected among all analyzed serotypes (Fig. 2B–H).

### AAV-mediated Rescue Experiment for the *Crx* KO Retina

**Figure 3:**
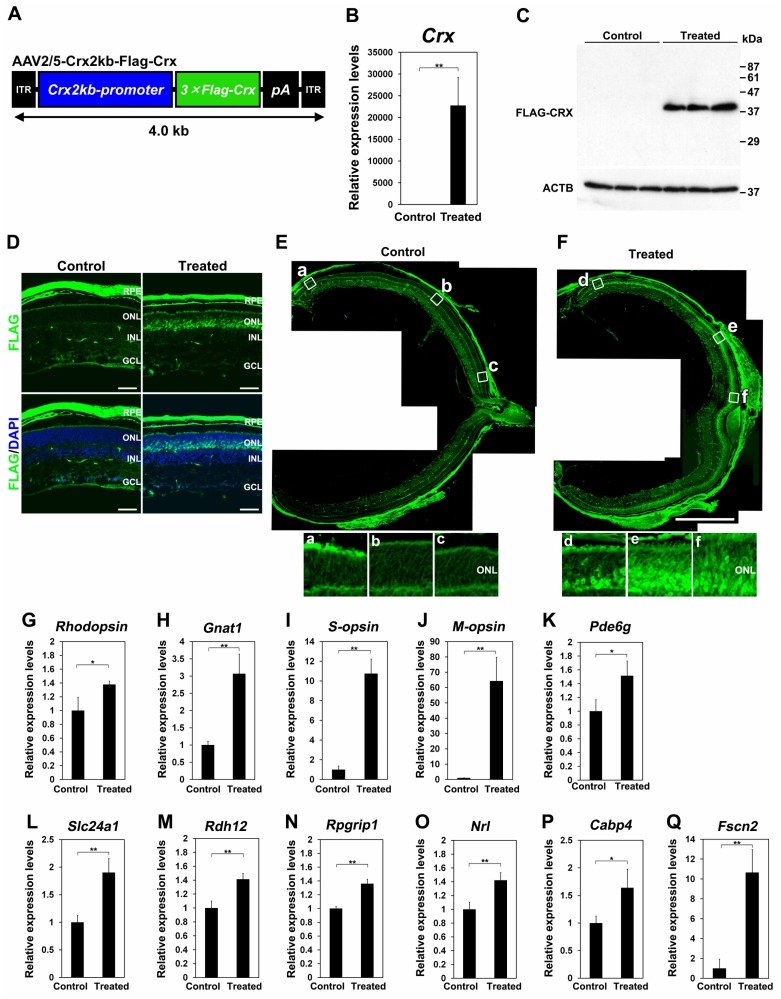
Gene expression analysis of the *Crx* KO retina treated with AAV-Crx. (A) Schematic diagram of the AAV2/5-Crx2kb-Flag-Crx construct. (B) Expression analysis of *Crx* by RT-qPCR using RNA isolated from control and AAV-treated *Crx* KO retinas (Control *Crx* KO retinas: n = 3 from three different mice and AAV-treated *Crx* KO retinas: n = 4 from four different mice). (C) Western blot analysis of FLAG-CRX using control and AAV-treated *Crx* KO retinas from three different mice respectively. An anti-FLAG antibody was used to detect FLAG-CRX. ACTB (β-actin) was used as a loading control. (D–F) Immunostaining of the *Crx* KO retinas treated with AAV-Crx or PBS using an anti-FLAG antibody. The distribution of FLAG-CRX expression in control and AAV-Crx treated *Crx* KO retinas (E, F). Enlarged images in white boxes (a-c and d-f) in Figure 3E and F, respectively. Scale bar represents 50 µm (D) and 1 mm (E, F). (G–Q) Expression analyses of eleven genes related to human retinal diseases three weeks after treatment (Control *Crx* KO retinas: n = 3 from three different mice and AAV-treated *Crx* KO retinas: n = 4 from four different mice). *Rhodopsin* (G), *Gnat1* (H), *S-opsin* (I), *M-opsin* (J), *Pde6g* (K), *Slc24a1* (L), *Rdh12* (M), *Rpgrip1* (N), *Nrl* (O), *Cabp4* (P), and *Fscn2* (Q). Control retinas were injected with PBS (Vehicle). *Rpl4* was used for normalization. Primers for qPCR were listed in Table S1. Error bar represents the SD from the means of three control retinas and four treated retinas. ^**^ *p*<0.01, ^*^ *p*<0.05. ITR: inverted terminal repeat, RPE: retinal pigment epithelium, ONL: outer nuclear layer, INL: inner nuclear layer, GCL: ganglion cell layer.

To validate the usefulness of AAV-mediated gene transfer into the developing mouse retina, we performed an AAV-mediated rescue experiment for the *Crx* KO mice. CRX is a transcription factor which plays a crucial role in photoreceptor maturation through photoreceptor gene transactivation [1], [8], [9], [10]. We generated an AAV2/5 vector expressing Flag-tagged *Crx* cDNA under the control of the *Crx 2kb* promoter to drive specific expression in photoreceptor cells (AAV2/5-Crx2kb-Flag-Crx = AAV-Crx) [17], [18] (Fig. 3A). We injected AAV-Crx subretinally into *Crx* KO retinas at P0. To confirm the *Flag-Crx* expression in the retina, we performed an RT-qPCR analysis using RNA from the whole retina at three weeks after injection. We observed significant expression of *Flag-Crx* mRNA in the *Crx* KO retinas treated with AAV-Crx (Fig. 3B). We further analyzed FLAG-CRX expression in control and AAV-Crx treated retinas by western blotting using an anti-FLAG antibody. We detected a 38 kDa FLAG-CRX band in the AAV-treated *Crx* KO retinal lysates (Fig. 3C). To determine whether FLAG-CRX is expressed in photoreceptor cells, we performed an immunostaining of the control *Crx* KO and AAV-Crx-injected *Crx* KO retinas with the anti-FLAG antibody. FLAG signals were predominantly detected in photoreceptor cells in AAV-Crx-treated *Crx* KO retinas (Fig. 3D). Non-specific signals were also detected in the RPE and blood vessels in the INL in both control and AAV-Crx-treated *Crx* KO retinas. This is very likely due to autofluorescence from the RPE and to the reaction of the anti-mouse secondary antibody to endogenous mouse antibodies in the blood vessels (Fig. 3D). AAV-Crx-mediated FLAG-CRX expression was widely distributed throughout the AAV-Crx-treated *Crx* KO retinas (Fig. 3E, F).

The expression profiling of the *Crx* KO retina using microarray identified a number of photoreceptor genes down-regulated in the *Crx* KO retina involved in phototransduction, ciliary function, transcriptional regulation of photoreceptor genes, and synaptic development [10], [19]. In humans, mutations of some of these gene homologues cause retinal diseases, including retinal degeneration, color blindness and night blindness (RetNet: https://sph.uth.tmc.edu/retnet/). Down-regulation of these genes is likely to underlie the phenotypes of the *Crx* KO retina. Thus, we performed an expression analysis of photoreceptor genes, which are down-regulated in the *Crx* KO retina and related to human retinal diseases, in AAV-Crx-treated *Crx* KO retinas. We performed RT-qPCR analyses on the following eleven genes: *Rhodopsin* (phototransduction, RP and Congenital stationary night blindness (CSNB)), *Gnat1* (phototransduction, CSNB), *S-opsin*, *M-opsin* (phototransduction, color blindness), *Pde6g* (phototransduction, RP), *Slc24a1* (phototransduction, CSNB), *Rdh12* (visual cycle, LCA and RP), *Rpgrip1* (ciliary function, LCA and CORD), *Nrl* (transcription regulation, RP), *Cabp4* (synaptic function, CSNB, LCA), and *Fscn2* (Cytoskeleton regulation, RP and macular dystrophy). In AAV-*Crx*-treated *Crx* KO retinas, we observed substantial up-regulation of *S-opsin*, *M-opsin*, and *Fscn2* (Fig. 3I, J, Q) and modest up-regulation of *Rhodopsin*, *Gnat1*, *Pde6g*, *Slc24a1*, *Rdh12*, *Rpgrip1*, *Nrl*, and *Cabp4* (Fig. 3G, H, K–P).

### Immunohistochemistry of *Crx* KO Retinas Treated with AAV-Crx

**Figure 4:**
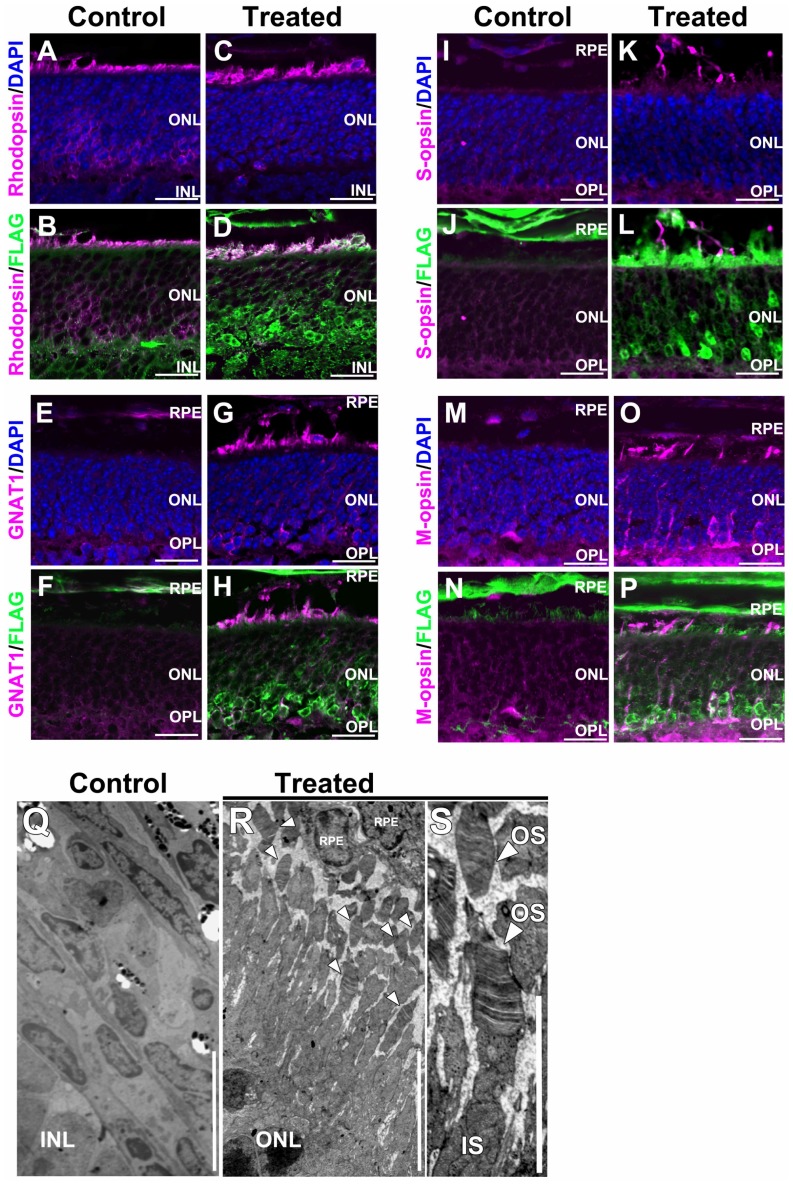
Histological analyses of the *Crx* KO retina transduced with AAV-Crx. (A–P) Immunostaining of the *Crx* KO retinas three weeks after AAV-Crx treatment with outer segment makers of rod photoreceptor (A–H) and cone photoreceptor cells (I–P). Scale bars represent 20 µm (A–P). (Q–S) Transmission electron microscopy analysis of retinas fifteen weeks after AAV-Crx treatment. (Q and R) In control retinas, no photoreceptor cell was observed (Q). In AAV-Crx-treated retinas, some outer segment structures were observed (R). (S) An enlarged image of outer segments in AAV-Crx-treated retinas. Arrowheads indicate outer segment containing disk lamina. Scale bars represent 7.5 µm (Q and R) and 5 µm (S). RPE: retinal pigment epithelium, ONL: outer nuclear layer, OPL: outer plexiform layer, INL: inner nuclear layer, GCL: ganglion cell layer. OS: outer segment, IS: inner segment.

We further analyzed the expression of RHODOPSIN, GNAT1, S-OPSIN, and M-OPSIN in AAV-Crx-treated *Crx* KO retinas by immunostaining. The RHODOPSIN protein level was slightly increased in AAV-Crx-injected *Crx* KO retinas (Fig. 4A–D), while GNAT1 signals were markedly increased (Fig. 4E–H). Similarly, S-OPSIN and M-OPSIN signals were markedly increased (Fig. 4I–P). Consistent with the results of the RT-qPCR analysis shown in Figure 3G–Q, the levels of these molecules also increased in AAV-Crx-treated *Crx* KO retinas. In addition, GNAT1, S-OPSIN, and M-OPSIN signals appeared to be localized in outer segments in AAV-Crx-injected *Crx* KO retinas (Fig. 4E–P). RHODOPSIN and GNAT1 are localized in the rod outer segment and S-OPSIN and M-OPSIN are localized in the cone outer segment. The *Crx* KO retina lacks outer segment formation [10], [20]. This immunohistochemical data suggests that the defect of outer segment formation in the *Crx* KO retina was partially restored by the subretinal injection of AAV-Crx into *Crx* KO retinas.

We further examined the morphology of the outer segment in the control *Crx* KO and AAV-Crx-injected *Crx* KO retinas in detail by transmission electron microscopy at fifteen weeks after AAV treatment (Fig. 4Q–S). We observed no photoreceptor cells in control retinas because of severe photoreceptor degeneration in the *Crx* KO retina (Fig. 4Q). In contrast, outer segments containing disk lamina were observed in AAV-Crx-injected *Crx* KO retinas (Fig. 4R, S). These data showed that the subretinal delivery of AAV-Crx into P0 *Crx* KO retinas partially restored outer segment formation in the *Crx* KO mice.

### The Improvement of Retinal Function in the *Crx* KO Retina Treated with AAV-Crx

**Figure 5:**
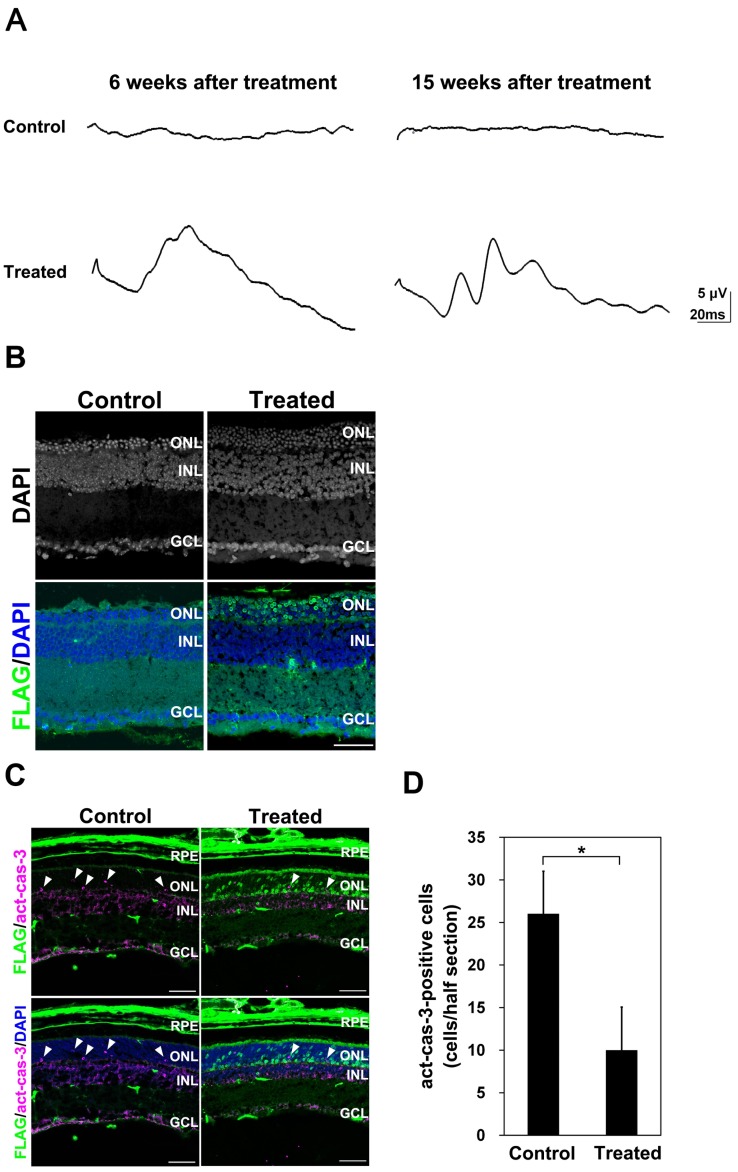
Restoration of function and morphology in the *Crx* KO retina treated with AAV-Crx. (A) Representative ERGs recorded at six and fifteen weeks after AAV-Crx treatment. ERG responses averaged from one hundred 1.0 log cd-s/m^2^ stimuli. The mice used for ERG recordings were independently prepared between the two time points. At both six and fifteen weeks after AAV treatment, all three of the control eyes from different mice showed no ERG response, while two of the three treated eyes from different mice showed significant responses. (B) Immunostaining of FLAG-CRX in the retinas at fifteen weeks after AAV-Crx treatment. (C and D) Immunostaining of active-caspase-3 and FLAG-CRX in *Crx* KO retinas three weeks after AAV-Crx treatment (C). The number of active-caspase-3-positive cells (D, Control retinas: n = 3 from three different mice and AAV-treated *Crx* KO retinas: n = 4 from four different mice). Arrowheads indicate active-caspase-3-positive cells. Scale bar represents 50 µm. Error bar represents the SD from the means of three control retinas and four treated retinas. ^*^ *p*<0.05. RPE: retinal pigment epithelium, ONL: outer nuclear layer, INL: inner nuclear layer, GCL: ganglion cell layer.

To evaluate the effect of the AAV-Crx injection on retinal function, we performed electroretinogram (ERG) recordings. *Crx* KO mice exhibit flat scotopic and photopic ERG responses at P30, resulting from the defect of formation of outer segments and considerable loss of phototransduction molecules in the *Crx* KO mice [10]. At 6 and 15 weeks after AAV treatment, we measured ERG responses from three control and three AAV-Crx-treated eyes. The mice used for ERG recordings were independently prepared between the two time points. We first tried to record scotopic ERG responses from the AAV-treated mouse eyes in a conventional way [21], [22], but no positive response was detected. Therefore, we then measured photopic ERG responses with one hundred stroboscopic flashes of 1.0 log cd-s/m^2^. The ERG responses obtained under these conditions majorly reflect cone photoreceptor functions. Consistent with our previous observation [10], all of the control eyes exhibited completely flat responses at both 6 and 15 weeks after AAV treatment (Fig. 5A top, Table 1). In contrast, two of three AAV-Crx-treated eyes showed significant photopic a- and b-waves at both 6 and 15 weeks after AAV treatment (Fig. 5A bottom, Table 1). One of the AAV-Crx-treated *Crx* KO mice did not show a detectable ERG photopic response. Although this mouse eye widely expressed FLAG-CRX, the eye was much smaller than the other two mouse eyes and was severely damaged histologically (data not shown), probably due to subretinal injection damage, resulting in physiological dysfunction. This result shows that the subretinal injection of AAV-Crx into *Crx* KO retinas partially restored the physiological function of *Crx* KO photoreceptor cells.

**Table 1:** Quantitative analysis of ERG amplitudes in control and AAV-Crx treated eyes.

	a-wave amplitudes	b-wave amplitudes
Control eye (6 weeks)		
1	0	0
2	0	0
3	0	0
AAV-treated eye (6 weeks)		
1	4.3	9.0
2	1.4	12.1
3	0	0
Control eye (15 weeks)		
1	0	0
2	0	0
3	0	0
AAV-treated eye (15 weeks)		
1	4.0	13.0
2	3.4	11.0
3	0	0

Progressive photoreceptor degeneration is also observed in the *Crx* KO mice [10]. We examined photoreceptor degeneration in the retinas fifteen weeks after AAV treatment by immunostaining using an anti-FLAG antibody (Fig. 5B). Although there was substantial photoreceptor cell death in both control and AAV-Crx-treated *Crx* KO retinas, AAV-Crx-treated *Crx* KO retinas had a thicker outer nuclear layer than that of control retinas (Fig. 5B top panels). Remarkably, most of the surviving photoreceptor cells in AAV-Crx-treated *Crx* KO retinas expressed FLAG-CRX (Fig. 5B bottom panels). This observation suggests that photoreceptor cells without FLAG-CRX expression died and that AAV-Crx inhibited photoreceptor cell death in the *Crx* KO retinas. To confirm this result, we performed immunostaining with an anti-active-caspase-3 antibody, an apoptosis marker, three weeks after AAV treatment (Fig. 5C, D). We observed a significant reduction of apoptotic cell numbers in AAV-Crx-treated *Crx* KO retinas (Fig. 5C, D). These results show that subretinal injection of AAV-Crx prevented photoreceptor cell death to some extent in the *Crx* KO retina.

## Discussion

The main goal of this study is to establish a method for AAV-mediated retinal gene transfer into developing retinas for *in vivo* analysis of retinal genes and to apply the method to the rescue of retinal degeneration. Through the transduction of AAV-CAG-mCherry into the developing mouse retina, we showed that various retinal cell types were transduced differently with each of seven serotypes of AAV. We quantitatively analyzed infection efficiencies of these AAV serotypes. In addition, we demonstrated the usefulness of AAV transduction into the developing mouse retina for the rescue of the retinal degeneration of *Crx* KO mice. These results will be useful to researchers who perform *in vivo* analysis of retinal genes using AAV.

We subretinally injected seven serotypes of AAV into the P0 mouse retina. We chose P0, because the retina at this stage is still developing and amenable to surgical treatment by subretinal injection. Serotypes AAV2/5 and AAV2/9 exhibited remarkable and efficient infection. Photoreceptor cells were selectively and efficiently transduced with AAV2/5. Retinal cells, except for Müller glial cells and bipolar cells, were efficiently transduced with AAV2/9. We observed efficient transduction into horizontal and/or ganglion cells with AAV2/1, AAV2/2, AAV2/8, AAV2/9 and AAV2/10. These data suggests that *in vivo* gene transfer by subretinal injection of AAV into the mouse retina enables us to analyze gene function in retinal cell types that are difficult or impossible to transduce by *in vivo* electroporation (cone photoreceptor, horizontal, and ganglion cells) [3]. To perform an analysis specifically in a certain cell type in the retina, a cell type-specific promoter of relatively short length for each cell type will need to be developed in the future.

In the current study, the infection efficiencies of AAV into bipolar and Müller glial cells were low in all tested serotypes, although AAV2/1 efficiently targeted Müller glial cells. These two retinal cells undergo differentiation at the latest stages during development [21]. In contrast, retinal cells, which are generated at embryonic stages or at early postnatal stages (photoreceptor, horizontal, amacrine and ganglion cells), were transduced with all analyzed serotypes. These results suggest that the AAV serotypes tested in this study are more infectious to differentiated retinal cells than to retinal progenitor or differentiating cells. Since more than 100 AAV serotypes have been isolated [22], AAV serotypes which are capable of efficiently infecting bipolar and Müller glial cells may be found in the future. Our observations on AAV tropism in the developing retina in the current study may expand the application of AAV for *in vivo* transduction to retinal cell types, such as cone photoreceptors, horizontal cells, and ganglion cells, which are barely transduced by *in vivo* electroporation.The AAV tropisms shown in the current study were different from those for the adult mouse retina in previous reports. In subretinal transduction into the adult retina AAV2/1 mainly targets RPE cells, and AAV2/2, 2/5, and 2/8 mainly target both RPE and photoreceptor cells [6], [7]. However, based on our results using P0 mice, each AAV serotype targeted several retinal cell types. This observation is consistent with the results reported by Surace *et al*. [5], in which they showed the difference of tropisms for transduction into fetal, neonatal and adult retinas using AAV2/1-, 2/2-, and 2/5-CMV-EGFP. There are two possible explanations for the shift of AAV tropism between the developing retina and the mature retina. The first is that the expression or abundance of AAV receptors in host retinal cells may change during retinal development. The second is that injected virus particles may more easily diffuse across the retina due to the smaller size of the P0 retina and dynamic cell migration during retinal development. The differences of AAV tropisms between neonatal and adult mice are also observed in the aorta, liver and kidney [4]. This suggests that the time point of transduction of an AAV vector during development is important to appropriately perform gene transfer to a cell type of interest.

We attached the *Crx 2kb* promoter to the AAV vector for the rescue experiment on the *Crx* KO retina. We previously reported that this promoter directs specific gene expression in developing and mature rod and cone photoreceptor cells [17], [18]. Expression analysis by RT-qPCR and immunohistochemistry showed improved expression of both rod-specific genes (*Rhodopsin* and *Gnat1*) and cone-specific genes (*S-opsin* and *M-opsin*) in AAV-Crx-treated *Crx* KO retinas (Fig. 3G–Q and 4A–P). This indicates that the *Crx 2kb* promoter successfully drove the *Crx* expression in both rod and cone photoreceptor cells. Thus the *Crx 2kb* promoter can be used with AAV to drive expression specifically in photoreceptor cells.

AAV-Crx modestly but significantly improved the photopic ERG responses, but we were not able to detect scotopic ERG responses in AAV-treated eyes. In order to detect relatively weak ERG responses in AAV-Crx-treated *Crx* KO eyes, we stimulated the mouse eyes with one hundred stroboscopic flashes (1.0 log cd-s/m^2^). However, under these conditions, it is very difficult to measure scotopic ERG responses because the intermittently repeated flashing lights abrogate the dark adaptation that is necessary for scotopic ERG recordings. Because of such technical difficulties, we were not practically able to measure scotopic ERG responses in our current study. AAV-Crx also partially restored outer segment formation in the *Crx* KO mice. This result not only confirms that AAV transduction into the P0 retina is a useful method for *in vivo* retinal gene transfer but also suggests that gene therapy for human retinal diseases caused by *Crx* mutations is possible. In addition, this is the first report on AAV-mediated rescue for mice with mutations of a transcription factor regulating photoreceptor development and related to human photoreceptor degeneration. *Nrl*, *Nr2e3*, and *Otx2* also play crucial roles in photoreceptor development through transcriptional regulation of photoreceptor genes [1], [23], [24], [25]. The mutations of these genes in humans are associated with several types of retinal degeneration (RetNet: https://sph.uth.tmc.edu/retnet/home.htm). Our current result suggests that human retinal degenerations caused by the mutations in these transcription factors can be restored by AAV-mediated gene therapy. This possibility will be examined by AAV-mediated rescue of mice with these gene mutations in the future.

## Materials and Methods

### Animals

For the evaluation of the tropism of AAVs *in vivo*, we used ICR mice (Charles River). The *Crx* KO mice were generated as described in our previous study [10]. All procedures conformed to the ARVO statement for the Use of Animals in Ophthalmic and Vision Research, and these procedures were approved by the Institutional Safety Committee on Recombinant DNA Experiments and the Animal Research Committee of Osaka Bioscience Institute (approval ID 10-401) and Institute for Protein Research, Osaka University (approval ID 24-05-0). Mice were housed in a temperature-controlled room with a 12-hour light/dark cycle. Fresh water and rodent diet were available at all times.

### Plasmid Constructs

For the production of AAV-CAG-mCherry and AAV2/5-Crx2kb-Flag-Crx, we constructed *pAAV-CAG-mCherry* and *pAAV-Crx2kb-Flag-Crx*, respectively. The *CAG* promoter used in this study was previously described [26]. To produce *pAAV-CAG-mCherry*, we initially constructed *pCAGGS-mCherry*. We cut *pAAV-U6-shLhx2-CMV-mCherry* [27] with *NheI* and *NotI* and inserted the *mCherry* fragment into *pCAGGS* digested *EcoRI* and *NotI* using a *NheI*/*EcoRI* linker. Finally, to produce *pAAV-CAG-mCherry*, we obtained the *CAG-mCherry* fragment by cutting *pCAGGS-mCherry* with *SalI* and *BglII*, and inserted its fragment into *pAAV-IRES-hrGFP* (Agilent technologies) digested with *NotI* and *BglII* using a *NotI*/*SalI* linker DNA. To produce *pAAV-Crx2kb-Flag-Crx*, we initially constructed *pCRX(2K)-Flag-Crx-βgal* [17]. The *Flag-mouseCrx* fragment was obtained by cutting *pcDNA3-Flag-Crx* [28] with *XhoI* and *XbaI*. We inserted it into *pCRX(2K)-βgal* digested with *KpnI* and *NheI* using an *XhoI*/*SalI* linker DNA and obtained *pCRX(2K)-Flag-Crx-βgal*. We next constructed *pCRII-bluntI-Crx2kb-Flag-Crx*. We first obtained the *Crx2kb-Flag-Crx* fragment by cutting *pCRX(2K)-Flag-Crx-βgal* with *SalI* and *XhoI*. We inserted it into *pCRII-bluntI* digested with *XhoI*, and obtained *pCRII-bluntI-Crx2kb-Flag-Crx*. Finally, to produce *pAAV-Crx2kb-Flag-Crx*, we cut *pCRII-bluntI-Crx2kb-Flag-Crx* with *NotI* and *XhoI*, and inserted its fragment using a *NotI*/*BglII* linker into *pAAV-IRES-hrGFP* (Agilent technologies) digested with *NotI* and *BglII*.

### AAV Production

AAV was produced by triple transfection of an AAV vector plasmid, an adenovirus helper plasmid, and an AAV helper plasmid (*pAAV2/1*, *pAAV-RC* [Agilent technologies], *pXR5*, *pAAV2/8*, *pAAV2/9*, *pAAV2/rh10*, and *pEEV2/11*) into AAV-293 cells by the calcium phosphate method. The cells were harvested at 72 hours after transfection, and lysed by four freeze-and-thaw cycles. The supernatant was collected by centrifugation, and treated with Benzonase nuclease (Novagen) to eliminate cellular DNA/RNA and excess plasmid DNAs. This virus preparation was used for subretinal administration. A titer of each AAV (in vector genomes (VG)/mL) was determined by qPCR using SYBR GreenER Q-PCR Super Mix (Invitrogen) and Thermal Cycler Dice Real Time System Single MRQ TP870 (Takara). The primers used for AAV titrations are listed in Table S1. The titers of all serotypes of AAV-CAG-mCherry used in this study were adjusted to approximately 2×10^12^ VG/mL. The titer of AAV2/5-Crx2kb-Flag-Crx used in the *Crx* KO rescue experiment is 2.6×10^12^ VG/mL.

### Subretinal Injection

Subretinal injection of AAV was performed as described elsewhere [3], [29]. P0 mice were anesthetized by chilling on ice, the eye was opened by cutting along the fused junctional epithelium where the two eyelids come together, and a small incision was made with a 30-gauge needle in the sclera near the junction with the cornea. 0.4 µL of an AAV preparation was injected into the subretinal space through the incision using an Ito micro syringe (Ito Corporation) with a 33-gauge blunt-ended needle under a dissecting microscope. Fast Green dye was added to AAV preparations at a final concentration of 0.1% as a tracer to confirm that the AAV preparations were injected into the subretinal space [29]. For histological analyses, we only used retinas in which the dye in the AAV preparation was confirmed to be evenly distributed and which were without severe damage caused by the injection process.

### Immunostaining

For immunohistochemistry, 14 µm thick retina sections were washed twice in phosphate-buffered saline (PBS), and permeabilized with 0.1% Triton X-100 (wt/vol) in PBS, then incubated with PBS containing 4% donkey serum (vol/vol) for 1 h to block samples. The samples were incubated with a primary antibody at 4°C overnight. After washing with PBS, these samples were incubated with secondary antibodies at 25°C for 1 hour. In the current study, we also used the following primary antibodies: anti-RHODOPSIN antibody (1∶10000, O4886, Sigma) as a rod photoreceptor cell marker, anti-S-OPSIN antibody (1∶500, sc-14363, Santa Cruz) as a cone photoreceptor cell marker, anti-CALB1 antibody (1∶1000, PC253L, Sigma) as a horizontal cell marker, anti-CHX10 antibody (1∶200, MBL) as a bipolar cell marker, anti-PAX6 antibody (1∶100, DSHB) as an amacrine cell maker, anti-BRN3B antibody (1∶100, sc-6026, Santa Cruz) as a ganglion cell maker, anti-S100β antibody (1∶100, S-2532, Sigma) as Müller glial cell marker, anti-GNAT1 antibody (1∶3000, sc-389, Santa Cruz), anti-M-OPSIN antibody (1∶500, AB5402, Chemicon), and anti-FLAG antibody (1∶1000, F1804, Sigma). The following secondary antibodies were also used: Alexa Fluor 488-conjugated anti-mouse IgG (1∶300, A11001, Invitrogen), Alexa Fluor 488-conjugated anti-rabbit IgG (1∶300, A11008, Invitrogen), Alexa Fluor 488-conjugated anti-goat IgG (1∶300, A11055, Invitrogen), Cy3-conjugated anti-mouse IgG (1∶300, 715-165-150, Jackson), and Cy3-conjugated anti-rabbit IgG (1∶300, 705-165-147, Jackson). For immunostaining of the whole retina, each retina was gently peeled off from the sclera, rinsed in PBS, and fixed with 4% paraformaldehyde (wt/vol) in PBS for 1.5 h. The retinas were permeabilized by incubation in 0.1% Triton X-100 in PBS (PBST) for 30 min. After washing in PBST, samples were blocked with 4% donkey serum in PBST for 1 h. The retinas were then immunostained with primary antibodies against mCherry (1∶1000, 632496, Clontech) at 4°C overnight. After washing in PBST, reactions with a Cy3-conjugated anti-rabbit IgG secondary antibody were performed overnight at 4°C.

### Quantification of Infection Efficiency

AAV-CAG-mCherry-injected retinas were co-immunostained with cell type-specific markers for each retinal cell shown above and an anti-mCherry antibody. We counted the number of marker-positive cells and marker/mCherry double-positive cells according to cell types for the calculation of infection efficiencies. We used high-resolution confocal images of retinal sections along the z-axis (2.0 µm) taken with the LSM 700 (Zeiss, 20× or 40× objectives) to count cell numbers and measure infection efficiencies for rod and cone photoreceptor, bipolar, Müller glial, and amacrine cells. Since horizontal and ganglion cells in the confocal images (20×) are small in number (<10 cells), it is very difficult to accurately calculate the infection efficiencies of these cell types in contrast to other cell types. Thus, we counted the whole cell numbers seen through a fluorescence microscope for calculating the infection efficiencies of these cell types. We used three retinas from three different mice and counted 100–200 rod photoreceptor and amacrine cell marker-positive cells, 30–45 cone photoreceptor cell marker-positive cells, 30–100 horizontal and ganglion cell marker-positive cells, 40–140 bipolar cell marker-positive cells, and 30–60 Müller glial cell marker-positive cells for measuring infection efficiency for each retinal cell type. To show that AAV infection diffused throughout the retina, infection efficiencies in rod photoreceptor cells were calculated in the three areas, which are central, middle and peripheral areas to optic nerve head at the side uninjured by injection. Infection efficiencies in cone photoreceptor, bipolar, amacrine, and Müller glial cells were calculated in the central area. Infection efficiencies in horizontal and ganglion cells were calculated throughout the retina of the side uninjured by injection.

### Western Blot Analysis

Western blot analysis was performed as described previously [27]. The membrane was incubated with an anti-FLAG antibody (1∶1000, F1804, Sigma). The membrane was then incubated with a horseradish peroxidase–conjugated goat antibody against mouse IgG (1∶10000, Zymed). For the secondary immunoreaction, the PVDF membrane was incubated with WB Stripping Solution (Nacalai Tesque) to remove antibodies, and blocked again with 5% skim milk (wt/vol) in TBS. Further immunoblots were performed using a mouse antibody against β-actin (ACTB, 1∶5000, Sigma).

### RT-qPCR

The mouse retinas were harvested and dissected at 3 weeks after injection. Total RNA (1 µg) from the retina was isolated using TRIzol reagent (Invitrogen) and converted to cDNA using Superscript II Reverse Transcriptase (Invitrogen). Real time PCR was performed using SYBR GreenER Q-PCR Super Mix (Invitrogen) and Thermal Cycler Dice Real Time System Single MRQ TP870 (Takara) according to the manufacturer's instructions. Quantification was performed by Thermal Cycler Dice Real Time System software version 2.0 (Takara). The primer sequences used for qPCR are listed in Table S1.

### ERG Recordings

Mice were anesthetized with an intramuscular injection of 80 mg/kg ketamine and 16 mg/kg xylazine. Pupils were dilated with topical 0.5% tropicamide and 0.5% phenylephrine HCl, and the mice were placed on a heating pad for the duration of the ERG recordings. ERGs were recorded with a gold wire loop placed on the cornea anesthetized with 1% tetracaine. A gold wire electrode was placed on the sclera 1 mm from the temporal limbus as the reference electrode. The mice were placed in a Ganzfeld bowl and 100 stroboscopic stimuli of 1.0 log cd-s/m^2^ (PS33 Plus; Grass Telefactor) were averaged with a repetition rate of 1 sec to record the ERGs. Signals were amplified and bandpass filtered between 1 and 1000 Hz (Power Lab; AD Instruments, Castle Hill, Australia). Amplitudes of both a- and b-waves were quantified from photopic ERG responses.

### Transmission Electron Microscopy

Specimens for transmission electron microscopy were prepared in the following manner. Eyes were enucleated from anaesthetized mice. Following the removal of anterior segment, each posterior eyecup was fixed with 2% glutaraldehyde and 2% paraformaldehyde in a cacodylate-based buffer adjusted at pH 7.4. After fixation with 1% osmium tetraoxide for 90 min, the retinas were dehydrated through a graded series of ethanol (50%–100%) and n-butylglycidylether. Finally, they were embedded in epoxy resin. Ultrathin sections were cut on an ultramicrotome (Ultracut E, Reichert-Jung, Vienna, Austria), and stained with uranyl acetate and lead citrate. Retinas were observed by transmission electron microscope (1200EX, JEOL, Japan).

### Statistical Analysis

Statistical significance was calculated with a Student's t test. A value of *p*<0.05 was taken to be statistically significant. Data are presented as means ± SD.

## Supporting Information

Table S1 **Primer sequences.** Primers for qPCR analysis.
(XLS)
